# High rate of loss to clinical follow up among African HIV-infected patients attending a London clinic: a retrospective analysis of a clinical cohort

**DOI:** 10.1186/1758-2652-13-29

**Published:** 2010-08-04

**Authors:** Sarah M Gerver, Tim R Chadborn, Fowzia Ibrahim, Bela Vatsa, Valerie C Delpech, Philippa J Easterbrook

**Affiliations:** 1 Academic Department of HIV/GU Medicine, King's College London School of Medicine at Guy's, King's College and St Thomas' Hospitals, Weston Education Centre, London SE5 9RJ, UK; 2 HIV & STI Department, Centre for Infections, Health Protection Agency, 61 Colindale Avenue, London NW9 5EQ, UK; 3 Department of Infectious Disease Epidemiology, Division of Epidemiology, Public Health and Primary Care, Imperial College London, St Mary's Campus, Norfolk Place, London, W2 1PG, UK

## Abstract

**Background:**

Long-term regular clinic follow up is an important component of HIV care. We determined the frequency and characteristics of HIV-infected patients lost to follow up from a London HIV clinic, and factors associated with loss to all HIV follow up in the UK.

**Methods:**

We identified 1859 HIV-infected adults who had registered and attended a London clinic on one or more occasions between January 1997 and December 2005. Loss to follow up was defined as clinic non-attendance for one or more years. Through anonymized linkage with the Survey of Prevalent HIV Infections Diagnosed and Health Protection Scotland, national databases of all HIV patients in care in the UK up to December 2006, loss-to-follow-up patients were categorized as Transfers (subsequently received care at another UK HIV clinic) or UKLFU (no record of subsequent attendance at any HIV clinic in the UK). Logistic regression analysis was used to identify factors associated with UKLFU for those both on highly active antiretroviral therapy (HAART) and not on HAART.

**Results:**

In total, 722 (38.8%) of 1859 patients were defined as lost to follow up. Of these, 347 (48.1%) were Transfers and 375 (51.9%), or 20.2% of all patients, were UKLFU. Overall, 11.9% of all patients receiving HAART, and 32.2% not receiving HAART were UKLFU. Among those on HAART, risk factors for UKLFU were: African heterosexual female (OR = 2.22, 95% CI: 1.11-4.56) versus white men who have sex with men; earlier year of HIV clinic registration (1997-1999 OR: 3.51, 95% CI: 1.97-6.26; 2000-02 OR: 2.49, 95% CI: 1.43-4.32 vs. 2003-2005); CD4 count of < 200 versus > 350 cells/mm^3 ^(OR = 1.99, 95% CI:1.05-3.74); and a detectable viral load of > 400 copies/ml (OR = 5.03, 95% CI: 2.95-8.57 vs. ≤ 400 copies/ml) at last clinic visit.

Among those not receiving HAART, factors were: African heterosexual male (OR = 3.91, 95% CI: 1.77-8.64) versus white men who have sex with men; earlier HIV clinic registration (2000-2002 OR: 2.91, 95% CI: 1.77-4.78; 1997-1999: OR: 5.26, 95% CI: 2.71-10.19); and a CD4 count of < 200 cells/mm^3 ^(OR: 3.24, 95% CI: 1.49-7.04).

**Conclusions:**

One in five HIV-infected patients (one in three not on HAART and one in nine on HAART) from a London clinic were lost to all clinical follow up in the UK. Black African ethnicity, earlier year of clinic registration and advanced immunological suppression were the most important predictors of UKLFU. There is a need for all HIV clinics to establish systems for monitoring and tracing loss-to-follow-up patients, and to implement strategies for improving retention in care.

## Background

The widespread availability of highly active antiretroviral therapy (HAART) has transformed the prognosis of HIV-infected patients over the past 10 years [1,2]. However, while there has been a marked reduction in HIV-related mortality and morbidity [2,3], additional challenges have emerged in the long-term management of HIV, such as drug toxicities [4-6], treatment failure due to poor adherence [7,8] and/or drug resistance [9] requiring regimen change, and increasing non-HIV-related morbidity and mortality [10-12]. As with any chronic illness, HIV patients require long-term, regular clinical follow up to monitor disease progression and optimal timing of initiation of HAART, assess response and adherence to antiretroviral therapy and associated adverse events, and address sexual health and HIV prevention needs [13]. Long-term retention is therefore an important component of HIV care, and high rates of loss to follow up would compromise the effective delivery of HIV care, as well as reliable documentation of mortality.

Rates of adherence [7], virological suppression [14] and survival [14,15] are the most commonly reported measures of the effectiveness of HAART management, but this applies only to patients who remain under follow up and in care. Although there have been several studies on losses to follow up from HAART programmes across sub-Saharan Africa [16-20], until recently there had been a paucity of information on losses to follow up from HIV care in high-income countries [21-26]. Most of these studies were conducted prior to the widespread use of HAART [23,25,26] or in research cohorts [27-29]. Furthermore, since many clinics do not have established systems for identifying patients who are lost to follow up (LFU), there is need for a greater understanding of the rates and reasons for loss to follow up to develop strategies to optimize retention.

We undertook an evaluation of the frequency of LFU and characteristics of patients who were LFU among adult HIV-infected patients attending a large HIV clinic in south London. We then matched those patients who were LFU with the national databases of all HIV patients receiving care in the UK to establish whether patients were LFU due to transfer to another clinic in the UK, or were lost to all follow up in the UK, and the factors associated with this.

## Methods

### Study population

HIV care and treatment in the UK is delivered through 227 specialist treatment sites, of which King's College Hospital is the eighth largest clinic nationally and the second largest in south London (personal communication Chau Cuong, Survey of Prevalent HIV Infections Diagnosed, Health Protection Agency). The clinic is based in south-east London, which has the highest rate of new HIV diagnoses in the UK, accounting for approximately 10% of all new HIV diagnoses in 2006 [30]. The clinic population is also highly ethnically diverse - of 500 new HIV diagnoses between 1998 and 2000, 54.7% were in black Africans (34.8% from eastern Africa, 32.8% from west Africa, and 32.4% from south-east Africa) and 9.1% in black Caribbeans.

### Identification and definition of loss to follow up

We identified all HIV-1 infected patients (≥ 18 years) who registered and attended the King's College Hospital HIV clinic on at least one occasion over a nine-year period (between 1 January 1997 and 31 December 2005), and reviewed their follow-up records up to 31 December 2006. The day, 1 January 1997, was identified as the initial date for the selection of the study population as by this date, HAART was widely available, and we wished to avoid the potential bias of availability of HAART on losses to follow up.

These patients were categorized as either LFU or current clinical attendees (CCAs). LFU was defined as non-attendance at the Kings College Hospital clinic for at least one year (up to 31 December 2006); at this clinic, routine follow up is in general every three months for patients on HAART, and every three to six months for those not yet on HAART. CCAs were defined as those patients who remained under King's College Hospital clinic follow up as of 31 December 2006.

Patients defined as LFU were then matched against two national registers of HIV patients receiving care in the UK: the Survey of Prevalent HIV Infections Diagnosed (SOPHID), which covers England, Wales and Northern Ireland; and the Health Protection Scotland database. The intention was to determine if these LFU patients had received care elsewhere in the UK between 1 January 1997 and 31 December 2006 (or 30 June 2007 for London SOPHID). Records were linked by matching on three criteria: soundex code of surname, sex and date of birth [31]. Additional matches were obtained through the use of less stringent criteria, called "fuzzy matching", using part-soundex code (first two characters of the four-character code) to overcome misspelling of surnames, and sex and date of birth. All data were anonymized to preserve the patients' identity, and their subsequent treatment locations were not identified.

For each LFU patient, we determined whether they had either subsequently registered and received care at another HIV clinic in the UK, and were therefore only LFU from King's College Hospital (Transfers), or had not registered at any other UK HIV clinic and were therefore lost to all clinical follow up in the UK (UKLFU). In order to ascertain whether UKLFU was due to unknown deaths (as reports on deaths for clinic attendees are based on passive reporting only), we also matched all LFU patients against the Office of National Statistics register of deaths in England, Wales and Northern Ireland for persons aged 60 years or younger, and through Health Protection Scotland using soundex code, sex and date of birth.

We matched our female UKLFU with the National Study of HIV in Pregnancy and Childhood using soundex code, date of birth, date of HIV diagnosis and country of birth in order to ascertain whether these patients may have been seen elsewhere for antenatal care subsequent to leaving the King's College Hospital HIV clinic. We also determined whether their HAART therapy was for the prevention of mother to child transmission alone. Finally, as a supplementary exercise, we reviewed the medical records for those defined as UKLFU to identify any documentation of intent to leave the UK, or other indications of reasons for LFU, and so further inform our proposed strategies for addressing UKLFU.

### Statistical analyses

Demographic and clinical information, including gender, risk group, ethnicity, serial CD4 cell count and viral load, HAART history and dates of all clinic visits, were obtained from the HIV clinic electronic database for all patients. We compared the clinical characteristics of patients LFU from King's College Hospital HIV clinic (Transfers) and the UK (UKLFU), with all current clinic attendees (CCAs) at the King's College Hospital HIV clinic using Chi square or Fisher's exact tests for categorical variables, and Kruskal-Wallis rank test for continuous variables. Univariate and multivariate logistic regression analyses were performed to identify factors associated with UKLFU versus CCAs. Key variables included in the models were: year of clinic registration; age at HIV diagnosis; gender; risk group; ethnicity; use of HAART; initial clinical stage and CD4 count at clinic registration and also prior to LFU (or 31 December 2006 for those who were CCAs); and viral load at last visit prior to LFU for those on HAART.

**Figure 1:**
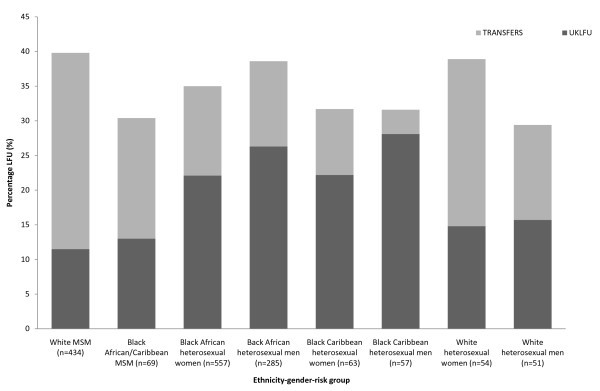
Percentage of Ethnicity-Gender HIV Risk groups LFU

We also repeated our analyses using a clinically relevant composite variable of gender-risk group and ethnicity, for descriptive (Figure 1 and Table 1) and adjusted analyses (Tables 2 and 3) to help identify the sub-group at greatest risk of UKLFU and to inform strategies to reduce UKLFU. Since we hypothesized that factors associated with LFU would differ according to whether patients were receiving HAART, analyses were further stratified according to whether they had been prescribed HAART within six months of their last clinic appointment prior to LFU, or at 31 December 2006 for those who were CCAs. All statistical analyses were performed using STATA 10.0 software (STATA Corp., Texas, USA).

**Table 1:** Characteristics of 1826 eligible HIV-positive King's College Hospital patients between 1 January 1997 and 31 December 2005 on ≥ 1 occasion^ǂ^

	CCAs	TRANSFERS	UKLFU	P value
	n = 1125*	n = 335*	n = 366*	
**Median age at HIV clinic registration (years) (IQR)**	33 (27-38)	30 (26-35)	31 (26-36)	< 0.001
**Gender-ethnicity risk group**	**n = 1119**	**n = 314**	**n = 352**	
White MSM	261 (23.3%)	123 (39.2%)	50 (14.2%)	
Black African/Caribbean MSM	48 (4.3%)	12 (3.8%)	9 (2.6%)	
Black African heterosexual female	362 (32.4%)	72 (22.9%)	123 (34.9%)	
Black African heterosexual male	175 (15.6%)	35 (11.2%)	75 (21.3%)	< 0.001
Black Caribbean heterosexual female	43 (3.8%)	6 (1.9%)	14 (4.0%)	
Black Caribbean heterosexual male	39 (3.5%)	2 (0.6%)	16 (4.6%)	
White heterosexual female	33 (2.9%)	13 (4.1%)	8 (2.3%)	
White heterosexual male	36 (3.2%)	7 (2.2%)	8 (2.3%)	
Other^†^	122 (10.9%)	44 (14.0%)	49 (13.9%)	
**Year of HIV clinic registration**				
1997-1999	219 (19.5%)	95 (28.4%)	103 (28.1%)	
2000-2002	353 (31.4%)	142 (42.4%)	158 (43.2%)	< 0.001
2003-2005	553 (49.2%)	98 (29.2%)	105 (28.7%)	
**Median baseline† CD4 cell count, cells/mm**^**3 **^**(IQR) cells/mm**^**3**^	289 (134-459)	322 (194-480)	348 (145-523)	0.001
Baseline** **CDC stage**	**n = 1125**	**n = 303**	**n = 322**	
Asymptomatic	734 (65.2%)	185 (61.1%)	224 (69.6%)	0.091
Symptomatic	232 (20.6%)	80 (26.4%)	61 (18.9%)	
AIDS	159 (14.1%)	38 (12.5%)	37 (11.5%)	
**Received HAART within 6 months of last visit**	834 (74.1%)	118 (35.2%)	125 (34.2%)	< 0.001
**Median CD4 cell count at last visit, cells/mm**^**3 **^**(IQR)**	410 (299-559)	376 (229-520)	368 (229-551)	< 0.001
**% with CD4 cell count < 200 cells/mm**^**3 **^**at last visit**	**n = 1112**	**n = 246**	**n = 289**	
	109 (9.8%)	48 (19.5%)	64 (22.2%)	< 0.001
**Median log viral load, copies/ml, at last visit (IQR)**	1.6 (1.6-2.3)	3.7 (1.7-4.7)	3.5 (2.0-4.3)	< 0.001
**% with VL ≤ 400 copies/ml at last attendance**	**n = 1109**	**n = 235**	**n = 266**	< 0.001
	855 (77.1%)	88 (37.4%)	90 (33.8%)	
**Median duration of clinic attendance (months)**	46.2 (26.3-72.3)	7.7 (1.2-24.3)	4.1 (0.7-19.1)	< 0.001
**Number of clinic visits in year prior to last clinic visit**	**n = 1114**	**n = 326**	**n = 362**	
≤ 2	25 (2.2%)	104 (31.9%)	149 (38.4%)	< 0.001

All percentages are column percentages; ǂ of the original 1,859 patients, 33 were deceased (9 UKLFU patients, 12 Transfers and 12 CCAs) and excluded from further analysis, leaving 1826 patients.

* Denominator values may differ due to missing data; † "other" gender-ethnic-risk group includes MSM and heterosexuals from other ethnic groups, such as Asian, black other, mixed (n = 122), and patients who acquired HIV through intravenous drug use (n = 82) and blood products/surgery (n = 6). ** Baseline refers to the first CD4 measurement and first reported CDC stage.

**CCAs**: current clinic attendees. **Transfers**: patients not seen at the KCH HIV clinic for at least 1 year, but who subsequently have been seen elsewhere for HIV treatment/care services in the UK. **UKLFU**: patients not seen at the King's College Hospital HIV clinic for at least 1 year, who have not been seen anywhere else for HIV treatment/care services in the UK for at least a year.

**Table 2:** Univariate & multivariate logistic regression for UKLFU vs. CCAs - on HAART (n = 959)

	n (%)*	Univariate OR (95% CI )	P value	Multivariate OR (95% CI)	P value
**Age at HIV clinic registration (per year older)**	959	0.99 (0.97-1.01)	0.308	-	-
**Gender-ethnicity risk group**	n = 956				
White MSM	205 (21.4%)	1.00 (REF)		1.00 (REF)	
Black African/Caribbean MSM	32 (3.4%)	1.41 (0.38-5.21)	0.605	1.48 (0.38-5.82)	0.575
Black African heterosexual female	327 (34.2%)	2.46 (1.32-4.58)	0.004	2.22 (1.11-4.56)	0.025
Black African heterosexual male	184 (19.2%)	2.66 (1.36-5.19)	0.004	1.31 (0.59-2.94)	0.505
Black Caribbean heterosexual female	27 (2.8%)	1.71 (0.46-6.37)	0.427	2.04 (0.49-8.46)	0.328
Black Caribbean heterosexual male	35 (3.7%)	2.27 (0.76-6.77)	0.140	2.25 (0.68-7.44)	0.186
White heterosexual female	25 (2.6%)	1.86 (0.50-6.98)	0.358	0.76 (0.09-6.46)	0.804
White heterosexual male	32 (3.4%)	2.53 (0.84-7.57)	0.098	2.70 (0.79-9.18)	0.113
Other	89 (9.3%)	1.92 (0.84-4.46)	0.123	1.76 (0.69-4.51)	0.237
**Year of KCH HIV clinic registration**	n = 959	0.82 (0.76-0.89)	< 0.001	0.81 (0.74-0.88)	< 0.001
2003-2005	403 (42.0%)	1.00 (REF)		1.00 (REF)	
2000-2002	320 (33.4%)	2.76 (1.69-4.51)	< 0.001	2.49 (1.43-4.32)	0.001
1997-1999	236 (24.6%)	3.28 (1.97-5.45)	< 0.001	3.51 (1.97-6.26)	< 0.001
**CDC stage prior to LFU**^**ǂ**^	n = 950				
AIDS	266 (28.0%)	1.00 (REF)		-	-
Symptomatic	400 (42.1%)	1.45 (0.86-2.44)	0.160	-	-
Asymptomatic	284 (29.9%)	1.24 (0.75-2.03)	0.405	-	-
**CD4 cell count prior to LFU**^**ǂ**^**, cells/mm**^**3**^	n = 945				
> 350	557 (58.9%)	1.00 (REF)		1.00 (REF)	
200-350	262 (27.7%)	1.39 (0.86-2.24)	0.174	1.64 (0.98-2.75)	0.060
< 200	126 (13.3%)	3.68 (2.25-6.03)	< 0.001	1.99 (1.05-3.74)	0.034
**Viral load prior to LFU**^**ǂ**^**, copies/ml**	n = 939				
≤ 400	825 (87.7%)	1.00 (REF)		1.00 (REF)	
> 400	114 (12.1%)	6.46 (4.09-10.21)	< 0.001	5.03 (2.95-8.57)	< 0.001

* N values are for the number of patients included in univariate analysis. ǂ or prior to 31^st ^December 2006 for CCAs

**Table 3:** Univariate & multivariate logistic regression for UKLFU vs. CCAs - NOT on HAART (n = 532)

	n (%)*	Univariate OR (95% CI )	P value	Multivariate OR (95% CI)	P value
**Age at HIV clinic registration (per year older)**	n = 532	0.99 (0.97-1.01)	0.308	-	-
**Gender-ethnicity risk group**	n = 515				
White MSM	106 (20.6	1.00 (REF)		1.00 (REF)	
Black African/Caribbean MSM	25 (4.9%)	0.61 (0.23-1.67)	0.340	0.58 (0.17-1.94)	0.373
Black African heterosexual female	158 (30.7%)	1.67 (1.00-2.78)	0.048	1.57 (0.82-2.98)	0.172
Black African heterosexual male	66 (12.8%)	4.17 (2.17-8.03)	< 0.001	3.91 (1.77-8.64)	0.001
Black Caribbean heterosexual female	30 (5.8%)	1.13 (0.48-2.62)	0.783	1.47 (0.55-3.90)	0.442
Black Caribbean heterosexual male	20 (3.9%)	2.38 (0.90-6.26)	0.080	2.16 (0.67-7.01)	0.199
White heterosexual female	16 (3.1%)	0.88 (0.29-2.74)	0.831	0.46 (0.09-2.38)	0.356
White heterosexual male	12 (2.3%)	0.65 (0.17-2.54)	0.534	1.91 (0.36-10.12)	0.448
Other	82 (15.9%)	1.68 (0.93-3.03)	0.086	1.48 (0.69-3.20)	0.318
**Year of KCH HIV clinic registration**	n = 532	0.78 (0.74-0.84)	< 0.001	0.75 (0.68-0.83)	< 0.001
2003-2005	255	1.00 (REF)		1.00 (REF)	
2000-2002	191	2.77 (1.88-4.09)	< 0.001	2.91 (1.77-4.78)	< 0.001
1997-1999	86	4.70 (2.78-7.94)	< 0.001	5.26 (2.71-10.19)	< 0.001
**CDC stage prior to LFU**^**ǂ**^	n = 495				
AIDS	31 (6.3%)	1.00 (REF)		1.00 (REF)	
Symptomatic	131 (26.5%)	0.40 (0.18-0.89)	0.025	0.83 (0.28-2.44)	0.732
Asymptomatic	333 (67.3%)	0.64 (0.30-1.33)	0.229	1.51 (0.52-4.41)	0.446
**CD4 cell count prior to LFU**^**ǂ**^**, cells/mm**^**3**^	n = 456				
> 350	306 (67.1%)	1.00 (REF)		1.00 (REF)	
200-350	103 (22.6%)	1.03 (0.65-1.64)	0.908	0.98 (0.58-2.44)	0.941
< 200	47 (10.3%)	3.55 (1.86-6.79)	< 0.001	3.24 (1.49-7.04)	0.003

* N values are for the number of patients included in univariate analysis. ǂ or prior to 31st December 2006 for CCAs

## Results

There were 1859 HIV-positive patients aged 18 years or older who had registered and attended the King's College Hospital HIV clinic on at least one occasion over a nine-year period (between 1 January 1997 and 31 December 2005). Overall, there were 625 (33.6%) black African or Caribbean women and 348 (18.7%) black African or Caribbean men, and 445 (23.9%) white men who have sex with men (MSM). Less than 5% were either white heterosexual men or women. The median age at HIV diagnosis was 32 years (IQR 27-37).

In all, 432 (23.2%) patients were registered at the King's College Hospital HIV clinic between 1997 and 1999, 666 (35.8%) between 2000 and 2002, and 761 (40.9%) between 2003 and 2005. The initial median CD4 cell count was 303 (IQR: 141-467). In total, 762 (40.1%) had been prescribed HAART within six months of their last clinic visit. Those prescribed HAART within six months of their last clinic visit were more likely to be white than those who had not received HAART, even after adjustment for CD4 count, but there was no significant difference in gender or risk group.

**Figure 2:**
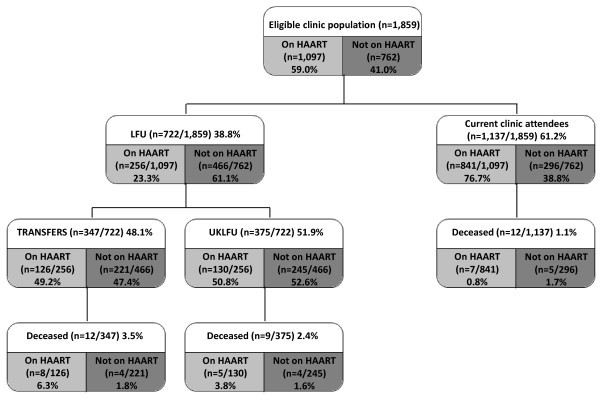
**Loss to follow-up among 1859 clinic patients **LFU defined as not seen for ≥ 1 year at the King's College Hospital HIV clinic (up to 31^st ^December 2006). Transfers defined as LFU patients who subsequently have been seen elsewhere in the UK. UKLFU defined as LFU patients not seen at any treatment site in the UK for ≥ 1 year. CCAs defined as patients who remained under King's College Hospitals clinic follow-up as of 31^st ^December 2006.

A total of 722 (38.8%) of 1859 patients were defined as LFU as they had not been seen at the clinic for a year or longer (23.3% and 61.1%, respectively, for those receiving and not receiving HAART; Figure 2). Of these 722, 347 (48.1%), or 18.7% of all registered patients, were defined as Transfers (49.2% and 47.4% of those patients LFU receiving or not receiving HAART, respectively) as they had subsequently been seen at another HIV clinic in the UK, and 12 of these (3.5%) had died.

In total, 375 (51.9% of 722 LFU patients, or 20.2% of the 1859 eligible clinic population) were defined as UKLFU because there was no record of any further clinical follow up in the UK (50.8% and 52.6% of those patients LFU receiving or not receiving HAART, respectively). Overall, nine (2.4%) had died. After exclusion of 33 deceased patients (12 CCAs, 12 Transfers and nine UKLFU), subsequent analyses were based on the remaining 366 UKLFU patients (125 on HAART and 241 not on HAART) apparently lost to all follow up in the UK, and therefore at the greatest risk of subsequent HIV-related morbidity and mortality.

### Frequency of LFU according to gender-ethnicity risk group

Figure 1 shows the percentages of Transfers (n = 326) and UKLFU (n = 360) according to eight ethnicity-gender HIV risk group categories. The highest percentage of Transfers to another HIV clinic in the UK was among white MSM (29.2%), followed by white women (24.1%), black African or Caribbean MSM (17.1%), and white heterosexual men (15.1%). In contrast, UKLFU was lowest among white and black MSM (11.2% and 14.3%, respectively), and white women (14.8%); it was highest among black Caribbean (29.3%) and African (26.2%) men, followed by black Caribbean and African women (23.4% and 22.1%, respectively).

### Characteristics of Transfers and UKLFU versus current clinic attendees

Table 1 compares the demographic and clinical characteristics of current clinic attendees with Transfers and UKLFU after exclusion of the 33 patients who were known to have died. There was a higher proportion of white MSM (39.2%) among Transfers, compared with UKLFU (14.2%) or CCAs (23.3%, p < 0.001), while UKLFU had a higher proportion of black African heterosexual men (21.3%) compared with Transfers (11.2%) or CCAs (15.6%). A greater percentage of CCAs first registered for HIV care at the Kings College Hospital HIV clinic between 2003 and 2005 than Transfers and UKLFU (49.2% vs. 29.2% and 28.7%, respectively, p < 0.001).

There were no significant differences between the groups in baseline clinical stage, but there was a statistically significant trend towards higher CD4 counts for UKLFU, Transfers and CCAs (348 vs. 322 vs. 289 cells/mm^3^, respectively). The UKLFU group had a much lower percentage receiving HAART than CCAs (34.2% vs. 74.1%, p < 0.001) and a corresponding lower CD4 count prior to loss to follow up (p < 0.001). The median duration of follow up was 46.2 months (IQR 26.3-72.3) for CCAs, 7.7 months (IQR 1.2-24.3) for Transfers and 4.1 months (IQR 0.7-19.1) for UKLFU (p < 0.001) (2.2 months (IQR 0-14.7) for those not on HAART, and 9.9 months of follow up (IQR 3.0-27.9) for those recently prescribed HAART). Overall, 38.4% (n = 149) of UKLFU patients had made two or less clinic visits in the year prior to becoming LFU (19.2% for those on HAART and 48.5% not on HAART) compared with 31.9% of Transfers (p < 0.001).

### Factors associated with LFU among those receiving or not receiving HAART

Overall, in a multivariate analysis, the factors most strongly associated with the UKLFU group versus the CCAs group were: not receiving HAART versus current HAART use (OR = 2.14, 95% CI: 1.14 to 3.27); being a black African heterosexual man (OR = 1.91, 95% CI: 1.08 to 3.35) or black African heterosexual woman (OR = 1.93, 95% CI: 1.18 to 3.15) versus white MSM; earlier clinic registration in 1997 to 1999 (OR = 4.81, 95% CI: 3.06 to 7.57) and 2000 to 2002 (OR = 2.90, 95% CI: 1.96 to 4.30) versus registration in 2003 to 2005; a CDC code of E1 (asymptomatic HIV) (OR = 1.92, 95% CI: 1.14 to 3.24); having a low CD4 count of < 200 versus > 350 cells/mm^3 ^(OR = 2.30, 95% CI: 1.14 to 3.24), and a detectable viral load of > 400 versus ≤ 400 copies/ml (OR = 3.86, 95% CI: 2.59 to 5.73) at last attendance.

After stratification according to HAART use, independent risk factors for UKLFU among those receiving HAART were: black African heterosexual female versus MSM (OR = 2.22, 95% CI: 1.11 to 4.56); earlier HIV clinic registration (1997-1999 OR = 3.51, 95% CI: 1.97 to 6.26; 2000-2002 OR = 2.49, 95% CI: 1.43 to 4.32) versus 2003-2005; a CD4 cell count of < 200 cells/mm^3 ^versus > 350 cells/mm^3 ^(OR = 1.99, 95% CI: 1.05 to 3.74); and a detectable viral load (OR = 5.03, 95% CI: 2.95 to 8.57) at last attendance.

Among those not receiving HAART, independent risk factors for UKLFU were: black African heterosexual male (OR = 3.91, 95% CI: 1.77 to 8.64); earlier clinic registration in 1997-1999 (OR = 5.26, 95% CI: 2.71-10.19) or 2000-2002 (OR = 2.91, 95% CI: 1.77 to 4.78); and a CD4 cell count of < 200 cells/mm^3 ^prior to loss to follow up (OR = 3.24, 95% CI: 1.49 to 7.04).

### Medical notes review

We reviewed the medical records of UKLFU for any documentation that might indicate reasons for being LFU in the UK, and so inform strategies to reduce loss to follow up. Of 366 UKLFU patients, 294 (80.3%) had their medical records located, and in 196 of these (66.7%), there was relevant documentation. Overall, in more than half (n = 115, 58.7%), there was an indication that they planned to leave the UK, either as a planned voluntary departure (n = 79, 40.3%) or because they were at risk of deportation because of documented immigration problems (n = 36, 18.4%). A further nine (4.6%) patients were documented to be in denial about their HIV status, and were not seen again following their initial HIV diagnosis.

## Discussion

In this first systematic study of losses to follow up in a UK HIV clinic, we found that 38.8% of 1859 HIV patients (23.3% on HAART and 61.1% not on HAART), who were registered at a large inner-city clinic over a nine-year period, discontinued their follow up at the clinic. Linkage with the national database of all HIV patients receiving care in the UK indicated that about half had transferred their care to another clinic in the UK, and the remaining half (20% of all registered patients; 11.9% of those patients receiving HAART, and 32.2% not receiving HAART) received no further HIV care in the UK. This percentage was highest among black African and Caribbean heterosexual men, with more than a quarter in these groups (26.2% and 29.3%, respectively) lost to all UK HIV care and follow up. Approximately one-third of those lost to all UK care were receiving antiretroviral therapy prior to being LFU, and only 2.4% of UKLFU could be attributed to deaths in the UK.

Our overall rate of one in five becoming lost to UK follow up (after exclusion of deaths) was higher than the 8.5% [21] and 11.9% [23] from two cohorts in France, but was comparable to the 25% reported by an Italian cohort with a high proportion of injecting drug users [26], the 27% reported by a Boston clinical cohort [22], the 22.0% reported by EuroSIDA (a clinical cohort encompassing 93 clinical centres in Europe, Israel and Argentina [24]), and the 16% reported in a survey of community-based settings led by the American Foundation of AIDS Research [25]. These differences were not explained by significant variations in definitions of LFU, as the majority defined LFU as patients who had not been seen in a clinic for at least 12 months [22-24,26-28].

However, the lower rates of LFU reported in one of the French cohorts [21] may be due to the shorter, one-year follow-up period compared with a cumulative LFU rate over a nine-year period in our study. Our overall high rate of loss to follow up also largely reflects the high proportion (more than 50%) of migrants originating from sub-Saharan Africa or the Caribbean in our clinic population, with high mobility and the highest rates of loss to follow up. The medical records review confirmed this, with documentation indicating that a high proportion planned to leave the UK, either voluntarily or because of immigration problems. In contrast, among white and black MSM, and white heterosexual women and men, the rates of UKLFU were less than 18%.

It is possible that we may have overestimated the proportion of HIV-infected patients LFU in the UK for several reasons. There may have been difficulties obtaining an exact match with the national SOPHID databases because patients may have registered at another site using a different date of birth or soundex code (either due to registration using different identifiers or due to coding differences, particularly in the transcribing and coding of uncommon surnames). In addition, the number of patients LFU who were deceased may have been underestimated since the national death register could only match deceased patients younger than 60 years of age. However, this would not have contributed significantly to a mismatch since only 10 of 366 (< 3%) UKLFU patients were older than 60 years.

Finally, we were unable to confirm whether patients UKLFU in the UK had in fact left the country and were either receiving ongoing care or had died in another country. In approximately half of those with available documentation, there was some indication that they either intended to leave the UK or that there were immigration problems, suggesting that they might have been deported. However, this information was not collected systematically, and was also available on only a subset of patients, and should therefore be interpreted with caution.

We found, overall, several independent risk factors associated with an approximately two-fold increased risk of being UKLFU: not receiving HAART; being a black African man or woman; registering for care prior to 2003; being asymptomatic; and having a CD4 count of less than 200 cells at last attendance. Our findings also show some similarities and differences in risk factors according to whether patients were receiving HAART or not at the time of defaulting from care. Common to both were an earlier clinic registration (before 2003) and a CD4 cell count < 200 cells/mm^3^. Among patients who had not received HAART, those who were black African heterosexual men were also more likely to be UKLFU, while among those on HAART, black African women and those with a detectable viral load were at increased risk of UKLFU.

These findings are consistent with those from several clinical or research cohorts in North America and Europe, which reported non-white ethnicity/migrants [21,22], younger age [22-24], a CD4 cell count of less than 200 cells/mm^3 ^[22,24] or a high CD4 count being associated with retention in care [25,26], non-use of HAART [24,27,28], a detectable viral load [21,22,24,27,28] or absence of an AIDS-defining illness as predictors of loss to follow up [24-26].

Therefore, among both HAART recipients and non-recipients, it is the most vulnerable patients who are most likely to be lost to follow-up. Vulnerable patients encompass those with advanced immunodeficiency, at the highest risk of disease progression and in need of ongoing care, as well as those on HAART, with poorly controlled viraemia with the added risk for onward HIV transmission of potentially HIV drug-resistant virus.

Our findings also show that black African men are the most likely to default from care before receiving HAART, consistent with studies from sub-Saharan Africa that have highlighted the challenges of engaging African men with HIV testing and clinical services [32-34]. In contrast, among those receiving HAART, the highest probability of loss to follow up was among African women (rather than men). Of note, this was not explained by pregnant women receiving short-term HAART for prevention of mother to child transmission and then defaulting, as this accounted for less than 10% of these women.

In addition, the median follow up for UKLFU was only 2.2 months for those not on HAART and 9.9 months for those recently prescribed HAART. This is similar to the findings from patient cohorts from France [23] and Italy [26], where the majority were LFU within six months of diagnosis. Nearly half of UKLFU patients not yet on HAART had made two or less clinic visits when they defaulted (usually following initial HIV testing and diagnosis). This highlights the importance of early supportive intervention in the weeks after initial diagnosis in prevention of early defaulting from care.

Several important measures to minimize LFU have been implemented at the clinic in the past 18 months. These include: text messaging reminders to patients' mobile phones the day before scheduled appointments; reducing the number of initial clinic visits with different members of the multi-disciplinary team following diagnosis; regular review of patients with missed appointments to allow early contact and intervention; and the appointment of a peer-support worker. However, formal prospective evaluation of the relative impact of these different strategies to reduce loss to follow up among HIV patients is now urgently needed in both developed and resource-limited settings.

A further important implication of our findings of high rates of loss to follow up is on the interpretation of outcomes, such as toxicity, rates of virological suppression, adherence and mortality, reported from clinical cohorts, which are based on those patients remaining in follow up, and may therefore substantially overestimate the proportion with a favourable outcome.

## Conclusions

We observed a high rate of loss to follow up from our HIV clinic in south London, and this was highest among black African men and women. Our results highlight the need to better understand the health-seeking behaviours of patients LFU and to implement strategies in HIV clinics for both better tracking and minimizing of loss to follow up from HIV care.

## Competing interests

The authors declare that they have no competing interests.

## Authors' contributions

SMG completed all the statistical analysis, wrote the first draft of the paper, and made alterations to the paper according to suggestions from the other authors. TRC with BP completed the matching of the patients LFU with the serial SOPHID databases. FI provided vital statistical support and advice to SMG. PJE conceived and designed the project, and with VCD, supervised the project and provided important advice and insight into the analyses and writing of the paper. All authors have had some involvement in the writing of the paper and have read and approved the final manuscript.

## Authors' information

Sarah Gerver has just completed a PhD in the Academic Department of HIV/GU Medicine, King's College London School of Medicine at Guy's, King's College and St Thomas' Hospitals. She is now a post-doctoral researcher, as an MRC Population Health Scientist Fellow in the Department of Infectious Disease Epidemiology at Imperial College London. Professor Philippa Easterbrook is Head of the Department of HIV/GU Medicine, King's College London School of Medicine at Guy's, King's College and St Thomas' Hospitals. Fowzia Ibrahim is a Statistican in the Academic Departments of Rheumatology and HIV/GU Medicine, King's College London School of Medicine at Guy's, King's College and St Thomas' Hospitals. Tim Chadborn is a Senior Scientist for SOPHID & CD4, Department of HIV and STIs, Health Protection Agency's Centre for Infections. Dr Valerie Delpech is Head of HIV Surveillance in the Department of HIV and STIs, Health Protection Agency's Centre for Infections. Bela Vatsa is a Scientist in the Department of HIV and STIs, Health Protection Agency's Centre for Infections.
